# A role for airway remodeling during respiratory syncytial virus infection

**DOI:** 10.1186/1465-9921-6-122

**Published:** 2005-10-21

**Authors:** David Becnel, Dahui You, Joshua Erskin, Dawn M Dimina, Stephania A Cormier

**Affiliations:** 1 Department of Biological Sciences, 202 Life Sciences Bldg., Baton Rouge, LA 70803, USA; 2 Deparment of Biochemistry & Molecular Biology, 13400 East Shea Boulevard, Scottsdale, AZ 85259, USA

**Keywords:** respiratory syncytial virus, pulmonary, inflammation, age factors, asthma, mice

## Abstract

**Background:**

Severe respiratory syncytial virus infection (RSV) during infancy has been shown to be a major risk factor for the development of subsequent wheeze. However, the reasons for this link remain unclear. The objective of this research was to determine the consequences of early exposure to RSV and allergen in the development of subsequent airway hyperreactivity (AHR) using a developmental time point in the mouse that parallels that of the human neonate.

**Methods:**

Weanling mice were sensitized and challenged with ovalbumin (Ova) and/or infected with RSV. Eight days after the last allergen challenge, various pathophysiological endpoints were examined.

**Results:**

AHR in response to methacholine was enhanced only in weanling mice exposed to Ova and subsequently infected with RSV. The increase in AHR appeared to be unrelated to pulmonary RSV titer. Total bronchoalveolar lavage cellularity in these mice increased approximately two-fold relative to Ova alone and was attributable to increases in eosinophil and lymphocyte numbers. Enhanced pulmonary pathologies including persistent mucus production and subepithelial fibrosis were observed. Interestingly, these data correlated with transient increases in TNF-α, IFN-γ, IL-5, and IL-2.

**Conclusion:**

The observed changes in pulmonary structure may provide an explanation for epidemiological data suggesting that early exposure to allergens and RSV have long-term physiological consequences. Furthermore, the data presented here highlight the importance of preventative strategies against RSV infection of atopic individuals during neonatal development.

## Introduction

Several experimental studies have shown a synergistic interaction between respiratory viral infections and allergic inflammation that exacerbate asthma [1-3]. RSV is the most common respiratory pathogen during infancy, and the majority of children worldwide have been infected with it by 2 years of age[4]. Several retrospective studies have suggested a link between RSV lower respiratory tract infections in infancy and the later development of asthma [5-10]. In a more recent study conducted by Sigurs and colleagues using a cohort of 140 children (47 of whom were hospitalized for RSV bronchiolitis during the first year of life and a control population of 93 infants with no history of RSV infection), it was found that by 7 years of age 30% of the children in the RSV group had experienced recurrent physician-diagnosed "wheeze" (ie, asthma) as compared to 3% of the control group[11]. Multivariant analyses further demonstrated that the greatest risk factor for asthma was RSV bronchiolitis independent of a family history of atopy. Moreover, the Tucson Children's Respiratory Study demonstrated that even children with mild RSV infections were 4 times more likely to have recurrent wheeze by 6 years of age[12]. Cumulatively, the data suggest that RSV bronchiolitis in infancy is associated with an increased risk of wheeze, which may persist for several years and is not adequately explained by a family history of atopy. Whether RSV directly contributes to the development of asthma remains unclear.

A recent study using an animal model of RSV infection demonstrated that "infections in early life play an important role" in shaping the secondary immune response to antigen leading to long-term consequences for the host[13]. In this study, Culley and colleagues showed that the age of initial infection played a significant role in the secondary response of these same animals to rechallenge with RSV at 12 weeks of age. Interestingly, if the mice were initially infected at 1 – 7 days of age, then their immune response to rechallenge at 12 weeks of age was characterized by enhanced bronchoalveolar lavage (BAL) cellularity including increased eosinophil and neutrophil numbers, increased CD8+ T cell numbers, and increased CD4+ T cell production of intracellular IL-4. The data seemed to indicate that a CD4+ response was important in creating the immune memory response of the neonatal mice to RSV, while a CD8+ response seemed important in the older animals (age 4 – 8 wks). Furthermore, these data suggested that the Th2 skewing of the immune response was likely to be important in eliciting disease pathogenesis. Although this work was seminal in demonstrating the importance of timing (7 d vs. 4 wks) of the initial infection on the subsequent T cell responses, it did not link these events with enhanced pulmonary dysfunction or demonstrate the prolonged contribution of these T cell responses to pathophysiological events involved in airway remodeling associated with asthma. Recently, Dakhama and colleagues[14] demonstrated that indeed timing of initial infection was a critical factor in determining the airway response to subsequent RSV infection. However, other risk factors for the development of pulmonary inflammation and wheeze (i.e., asthma) in humans exist and the enhancement of pulmonary inflammation by RSV may be dependent on the individual's atopic background and current exposure to allergens or other environmental factors.

Our hypothesis was that early exposure to RSV and allergen act synergistically to illicit inflammatory responses and long-term functional changes in the developing lung. We further hypothesized that these changes were the result of changes in airway structure (i.e., remodeling) and therefore, would compromise adult lung function. In the present study, weanling mice were exposed to RSV and/or ovalbumin (Ova) to examine the effect of early exposures on pulmonary pathophysiology. We report that weanling mice infected with RSV then exposed, Ova fail to develop airway hyperreactivity (AHR) or long-term pathophysiologic changes, while weanling mice exposed first to Ova then infected with RSV developed increased AHR and long-term pulmonary pathologies. This increase in AHR was accompanied by pulmonary inflammation due to increased eosinophil and lymphocyte cell numbers, mucus cell hypertrophy, and enhanced mucus production. Intriguingly, the mice also exhibited signs of airway remodeling including subepithelial fibrosis. The observed remodeling events were correlated with increased levels of various cytokines including TNF-α, IFN-γ, IL-5, and IL-2. Collectively, these data demonstrate that RSV infections, when combined with an allergic predisposition, can have long-term consequences for the lung and may contribute to the development of inflammatory disease states, such as asthma.

## Methods

### Mice

BALB/cJ mice, 6 – 10 weeks of age, were purchased from Jackson Labs and were maintained in ventilated micro-isolator cages housed in a specific pathogen-free animal facility. Sentinel mice within this animal colony were negative for antibodies to viral and other known mouse pathogens. All animal protocols were prepared in accordance with the Guide for the Care and Use of Laboratory Animals (National Research Council, 1996) and approved by the Institutional Animal Care and Use Committees at Mayo Clinic Arizona and Louisiana State University.

### Viral Preparation and Infection of Mice with RSV

**Figure 1:**
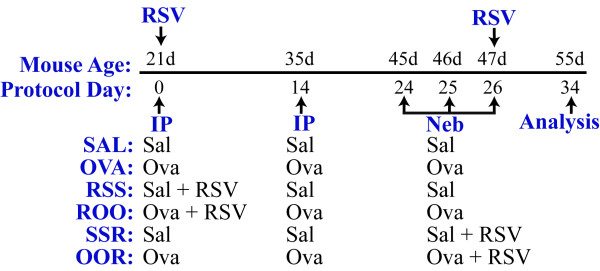
**Schematic of study protocol and exposure groups**. Weanling mice (21 days of age) were injected i.p. with ovalbumin complexed to Imject Alum (Ova, ROO, and OOR groups) or with isotonic saline (Sal, RSS, and SSR groups) on protocol days 0 and 14. After 1 h, the ROO and RSS groups were infected with RSV (10^4 ^TCID_50_/g body weight). These mice were then exposed to aerosolized ovalbumin or saline for 20 minutes on protocol days 24, 25, and 26. On protocol day 26, a subset of Sal and Ova mice were infected with RSV at 10^4 ^TCID_50_/g body weight (SSR and OOR groups, respectively).

RSV strain A2 (a kind gift of Dr. Barney Graham; NIH) was originally provided by Dr. R Chanock (NIH) and has since been maintained in culture by passage in HEp-2 cells. Master stocks and working stocks of virus were prepared as described elsewhere[15]. Prior to infection all mice were anesthetized with 3% isoflurane. Mice were subsequently infected intratracheally (i.t.) with RSV (10^4 ^TCID_50_/g body weight) or mock infected (i.e., culture media alone) at 21 or 47 days of age (Figure 1). Four days post-infection viral titer was determined using the TCID_50 _method of Spearman-Kärber[16,17].

### Ovalbumin Sensitization and Challenge Protocol

Mice were sensitized and challenged with chicken ovalbumin (Ova; crude grade IV; Sigma, St. Louis, MO) as previously described [18]. Mice were sensitized with an intraperitoneal (i.p.) injection of 0.1 ml (20 μg) Ova complexed with 2 mg Imject Alum (Al [OH]_3_/Mg [OH]_2_; Pierce, Rockford, IL) on protocol days 0 and 14 (Figure 1). Mice were subsequently challenged with an aerosol generated from an Ova solution (1% Ova w/v in saline) for 20 minutes on protocol days 24, 25, and 26 using an ultrasonic nebulizer (DeVilbiss, Somerset, PA). Control animals were injected i.p. with saline on days 0 and 14 and challenged with aerosolized saline on protocol days 24, 25, and 26 as described above. The mice were assessed for pulmonary cellular infiltrates, histopathologies, and lung function on protocol day 34.

### Study Protocol

The study protocol is outlined in Figure 1. The mice were divided into six groups. The SAL group was mock-allergen sensitized and challenged. The OVA group was Ova sensitized and challenged. The RSS group was mock-allergen sensitized and challenged and RSV-infected at 21 d of age. The ROO group was Ova sensitized and challenged and subsequently RSV-infected at 21 d of age. The SSR group was mock-allergen sensitized and challenged and subsequently RSV-infected at 47 d of age. The OOR group was Ova sensitized and challenged and subsequently RSV-infected at 47 d of age. Three mice in each group were sacrificed four days post-infection to assess pulmonary viral titers as described above. Assessment of airway reactivity and collection of tissue and BAL samples, as detailed below, were performed on protocol day 34 (55 days of age and 8 days following RSV infection).

### Assessment of Airway Reactivity in Response to Methacholine

#### Whole body plethysmography

Airway responsiveness to methacholine (MeCh), a muscarinic agonist, was assessed by whole body plethysmography (Buxco Electronics, Troy, NY and EMKA Technologies, Falls Church, VA) as described previously[19]. Mice were exposed for 3 minutes to aerosolized saline and subsequently to increasing concentrations of aerosolized MeCh (0, 3.125, 6.25, 12.5, 25, and 50 mg/ml in isotonic saline; Sigma). Following each nebulization, data including minute volume, tidal volume, breathing frequency, and enhanced paused (Penh) were recorded for 3 minutes. The Penh values measured during each 3-minute sequence were averaged and expressed for each dose of MeCh. Baseline Penh values did not differ significantly between any of the groups.

#### Invasive measurements of respiratory mechanics

Pulmonary resistance was measured using the forced oscillation technique as previously described[20]. Anesthetized animals were mechanically ventilated with a tidal volume of 10 ml/kg and a frequency of 2.5 Hz using a computer-controlled piston ventilator (Flexivent, SCIREQ; Montreal, Canada). Responses were determined in response to increasing concentrations of aerosolized MeCh (0, 1.875, 3.75, 7.5, 15, and 30 mg/ml in isotonic saline). All data that did not result in a coefficient of determination that was greater than 0.9 were excluded. The average value for each dose was calculated; and the percent difference from baseline per dose was then plotted.

### Sample Collection

On protocol day 34, all mice were euthanized and the following samples were obtained:

#### Lavage fluid

Bronchoalveolar lavage (BAL) of the lungs was performed using 1 ml of PBS containing 2% FBS. Total BAL cellularity was determined with the use of a hemocytometer. Cytospin slides were fixed and stained using the Diff-Quick kit (IMEB, Chicago, IL) and differential cell counts by unbiased observers were based on counts of 200–300 cells using standard morphological criteria to classify individual leukocyte populations. Four mice from each group were used for these analyses.

#### Pulmonary histology

Lungs were inflated with 1 ml of 10% neutral-buffered formalin via a tracheostomy tube. After instillation of fixative, the trachea was ligated, and the lung was excised and fixed in formalin for 24 hours at 4°C. These tissues were then embedded in paraffin, cut in 4 μm frontal sections, mounted onto slides, and stained with either hematoxylin and eosin (H&E), periodic acid-Schiff (PAS) to quantitate mucus, Masson's trichrome (MT) to quantitate airway collagen deposition, toludine blue to quantitate mast cells, or anti-MBP (major basic protein) antibodies to specifically evaluate tissue-infiltrating eosinophils as previously described[21]

#### Morphometric analyses

The MT data were analyzed morphometrically by digital image analysis using Image-Pro Plus software (version 5.0.1, Media Cybermetics, Inc., Silver Spring, MD). The following calculation was used to determine airway mucus: % airway mucus = (the area of airway epithelium staining positive for mucus/the total area of airway epithelium) × 100. To calculate the thickness of collagen deposition within the basement membranes, a random starting point was chosen and a single measurement was made between two points on either side of the collagen deposition at right angles to a tangent marking the perimeter of the basement membrane. For each airway, measurements at approximately 50 μm intervals from a randomly chosen starting point were made around the entire airway. The measured values were averaged for the airways of each animal and the mean values for each group were determined.

### Cytokine Assays

Cytokine levels in whole lung homogenates were determined using the Mouse Th1/Th2 Cytokine Cytometric Bead Array Kit (BD Biosciences) as per the manufacturer's instructions. The sensitivity of the assay was as follows: TNF-α – 6.3 pg/ml, INF-γ – 2.5 pg/ml, and IL-5, IL-4 and IL-2 – 5 pg/ml. The data was resolved in the FL3 channel and acquired with a BD FACScan™ flow cytometer. Data analyses were performed using the BD Cytometric Bead Array Software to generate standard curves for each cytokine and to determine sample cytokine levels.

### Statistical Analysis

Data are presented as mean ± SEM obtained from experiments with n = 8 for whole body plethysmography analysis of AHR, n = 4 for invasive measurements of pulmonary mechanics, pulmonary viral titers, and histology, n = 3 for cytokine assays. For AHR and BAL cellularity, differences between groups were evaluated by means of two-way ANOVA. Bonferroni post-tests were performed to compare between pairs of groups. To determine statistical significance of the morphometric data, we employed a Kruskal-Wallis test with a Dunn's post-test. A one-way ANOVA was used to compare the mean cytokine levels among the various groups followed by the Tukey-Kramer multiple comparisons tests for significance between the groups. This was repeated for each individual cytokine. Differences between means were considered significant when p < 0.05.

## Results

### AHR is Enhanced in Weanling Mice Exposed First to Ova then Infected with RSV

**Figure 2:**
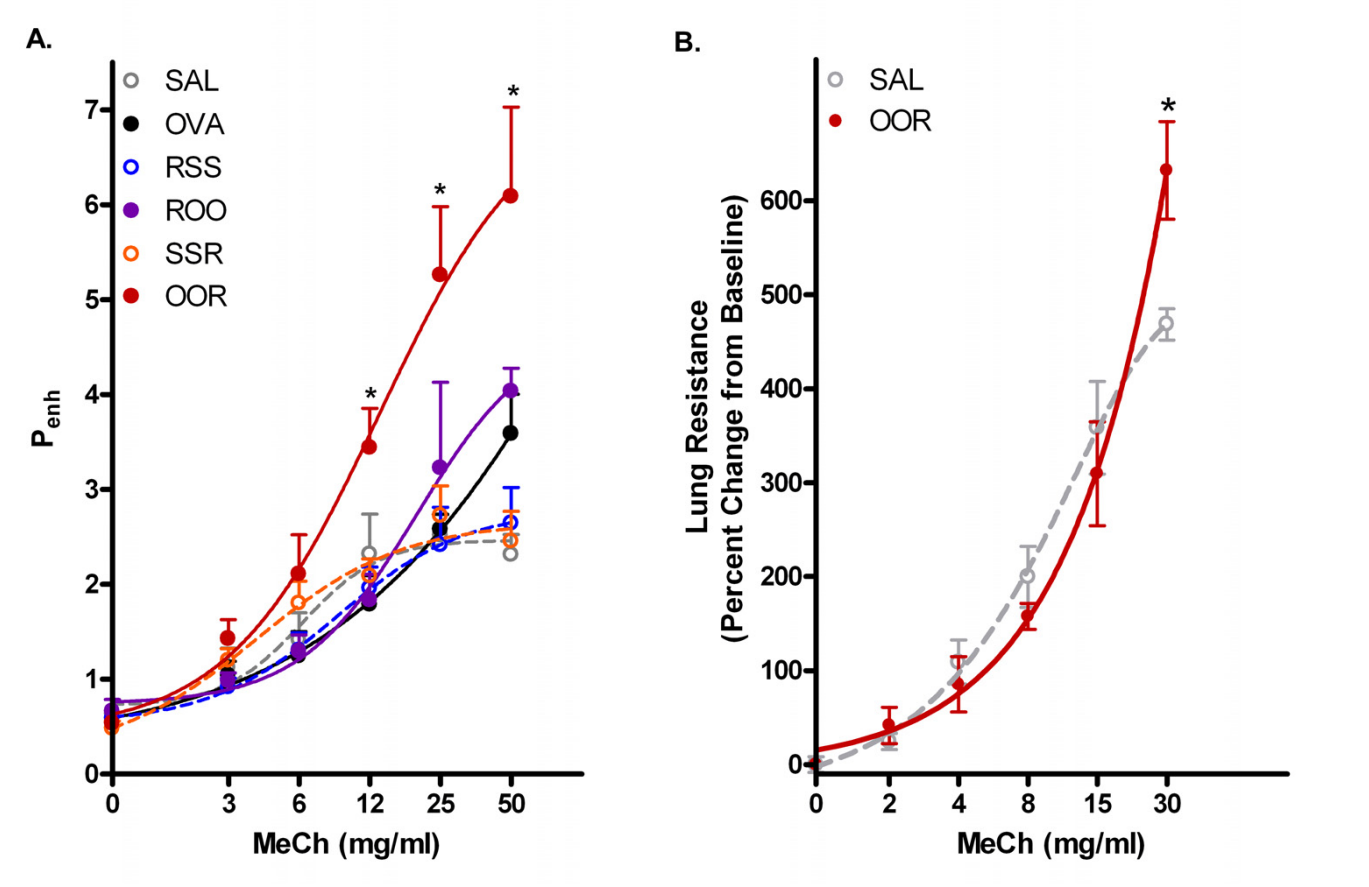
**Enhanced airway hyperreactivity and pulmonary resistance in weanling mice exposed to RSV and OVA**. A) Airway hyperreactivity (Penh) of each group is plotted as a function of increasing doses of inhaled MeCh. Data points are mean ± SEM from 8–10 mice per group. Groups as outlined in Figure 1. B) Lung resistance values were obtained by a forced oscillation technique and are plotted as a function of increasing doses of inhaled MeCh. Values presented are means ± SEM (n = 4 mice/group). *p < 0.001 for OOR vs. all other groups.

In order to investigate the role of RSV infection in relation to other environmental factors such as allergen, we sensitized and challenged weanling mice with Ova as shown in Figure 1. One hour post-sensitization, a subset of mice was infected with RSV (10^4 ^TCID_50_/g body weight). On the last day of allergen challenge, another subset of mice was infected with RSV. Eight days following the last Ova challenge, mice were exposed to increasing doses of MeCh and AHR was assayed. As shown in Figure 2A, RSV infection before (RSS) or after (SSR) saline administration failed to alter AHR. However, weanling mice exposed to Ova and then RSV (OOR) had significantly greater airway hyperresponsiveness to MeCh compared to all other groups (p < 0.001). AHR of weanling mice exposed first to RSV and then Ova (ROO) was similar to that of mice exposed only to Ova (OVA).

The consequences of altered airway responsiveness were further examined in vivo by invasive measurements of airway resistance. Respiratory system mechanics were assessed using the single-compartment model. The lung mechanics of weanling mice exposed to Ova and RSV (OOR) were significantly elevated as compared to control mice in response to MeCh administration (Figure 2B; p < 0.001).

### Allergen Sensitization and Challenge Decreases Pulmonary RSV Titer

Four days post-infection, we assessed pulmonary viral titers for all groups of mice. As expected, mice from the OVA and SAL groups displayed no evidence of viral replication. The mean viral titer in the lungs of weanling mice exposed to RSV alone (RSS) was 3.2 ± 0.08 log_10 _TCID_50_/g of lung, while viral titers in adult mice exposed to RSV (SSR) were 5.3 ± 0.1 log_10 _TCID_50_/g. Intriguingly, the reverse was true for mice infected with RSV in the presence of allergen sensitization. If mice were infected with RSV prior to Ova sensitization and challenge (ROO), then viral titers were high (5.2 ± 0.08 log_10 _TCID_50_/g); however if mice were sensitized and challenged with Ova prior to RSV infection (OOR) then the resulting viral titer (3.2 ± 0.2 log_10 _TCID_50_/g) was significantly lower (p < 0.001).

### BAL Cellularity Increases in Mice Exposed to Ova and RSV

**Figure 3:**
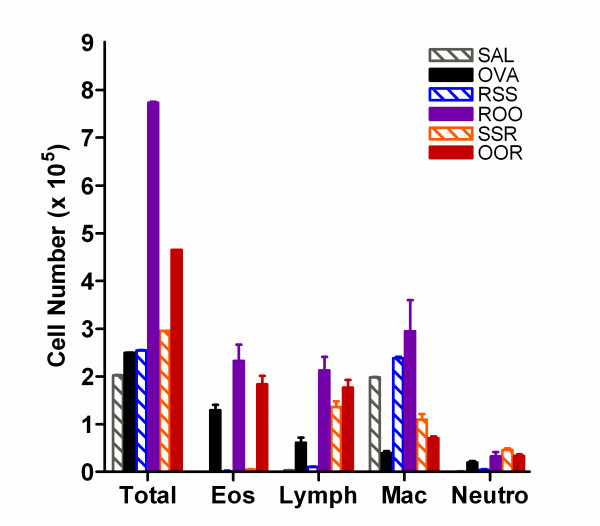
**BAL fluid cellularity is altered in mice exposed to RSV and/or OVA**. Differential cell counts were performed on Diff-quick stained cytospin preparations by two unbiased observers counting > 300 cells per sample. BAL cellularities are expressed as the product of the total number of cells recovered and the percentages of each cell type derived from differentials. Data are expressed as means ± SEM.

To evaluate the pulmonary immune response to RSV and Ova exposure, BAL fluid cells were recovered, and the total cellularity and composition of leukocytes among the groups of animals were compared. Total cell counts from the BAL fluid of the SAL, OVA, RSS, and SSR groups were not significantly different as shown in Figure 3. However, total cell counts from the BAL fluid of mice exposed to both RSV and Ova (ROO and OOR) were significantly greater than any other group (7.73 × 10^5 ^± 0.19 and 4.65 × 10^5 ^± 0.25; respectively). A comparison of individual cell numbers per ml of BAL fluid reveals that these increases were mainly due to increases in eosinophil and lymphocyte cell populations. To compare cell proportions among the different exposure groups, BAL cellularities were expressed as the product of the total number of cells recovered and the percentages of each cell type derived from differentials. RSV infection, in the presence of allergen challenge, led to a significant increase in total BAL cellularity (ROO and OOR). Interestingly, this increase was highest in the group of mice that were infected prior to allergen challenge (ROO). A significant reduction in the number of BAL macrophages was observed in the OVA, SSR, and OOR groups. Eosinophilia was observed only in the groups that received Ova (OVA, ROO, and OOR groups; p < 0.001). No significant difference in neutrophil numbers was observed in any of the groups.

### Enhanced Pulmonary Pathology is Observed in Weanling Mice Exposed to Ova and Subsequently Infected with RSV

**Figure 4:**
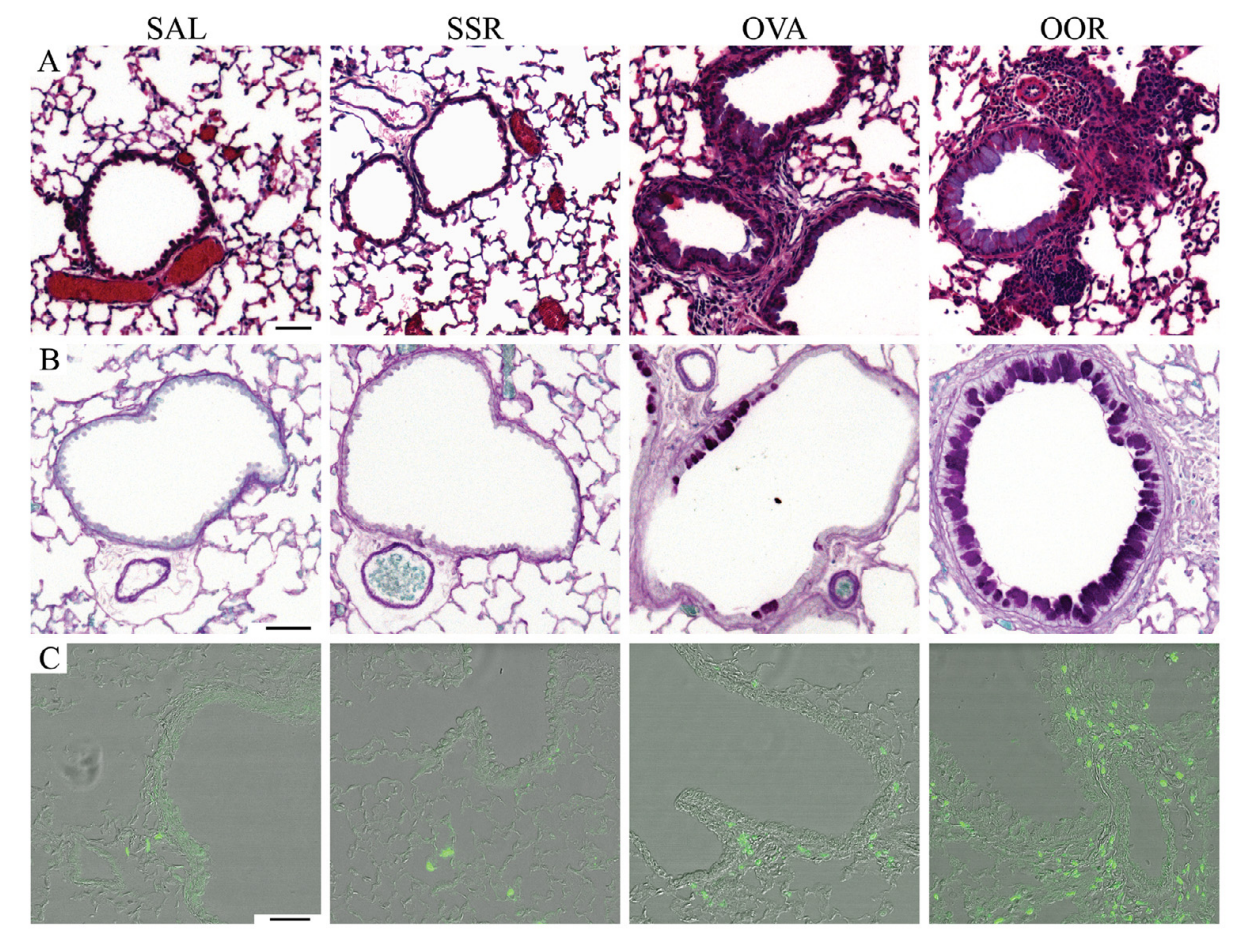
**Enhanced pulmonary histopathology is observed in mice exposed to Ova and RSV**. Lung sections from formalin-fixed, paraffin-embedded tissue were stained for A) cellularity, B) mucus (purple), and C) eosinophils (green) as described in the materials and methods section. The photographs are representative of the staining that occurs in the bronchioles of SAL, OVA, SSR, and OOR exposed mice. Although not shown, the RSS resembled the SSR group and the ROO group was not significantly different from the OVA mice. Scale bar = 50 μm.

The pulmonary histology induced by allergic sensitization and RSV infection is illustrated in Figure 4. Weanling mice exposed to Ova followed by infection with RSV (OOR) exhibited a significantly greater degree of pulmonary inflammation in both the peribronchial and perivascular regions (Figure 4, panel A). Additional histological analyses of the lungs of OOR mice demonstrated that other changes were present. Lung sections stained with PAS to detect mucus showed a significant increase in mucus production and mucus cell hypertrophy (Figure 4, panel B). Morphometric analyses of the PAS stained lung sections revealed a 2.3 fold increase in the percentage of airway mucus in the OOR group compared to the Ova group (35 ± 0.4% vs. 15 ± 2.2%, respectively; p < 0.05). Immunofluorescent staining using an anti-MBP antibody, which is specific for eosinophils, demonstrated an increase in the number of eosinophils that were associated with areas of expanded bronchus-associated lymphoid tissue (BALT) (Figure 4, panel C). There was no evidence of pulmonary inflammation, eosinophilia, or mucus production in the SAL, RSS, or SSR groups. In addition, no significant recruitment (i.e., average of 2 per entire lung section) of mast cells was observed in any of the exposure groups.

**Figure 5:**
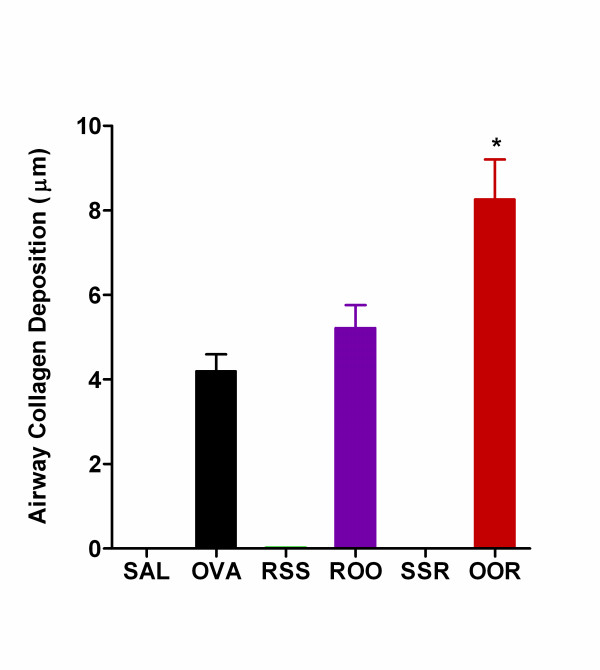
**Airway remodelling is evident in weanling mice exposed to Ova and RSV**. Fibrosis and deposition of collagen was observed in the subepithelial, reticular layer of the airways in the OVA, ROO, and OOR mice. Morphometric analyses using data collected at 50 μm intervals over the entire basement membrane revealed that these differences were significant. (8–10 measurements at ~50 μm intervals were collected for at least 5 airways; n = 4 mice per group). ROO vs. OOR, *p < 0.05 and OVA vs. OOR, *p < 0.01.

Visual analysis of the OOR lung sections indicated thickening of the subepithelial reticular layer indicative of airway remodeling. To investigate the role of airway remodeling in the enhanced pulmonary pathophysiology observed in these groups lung sections were stained with Masson's trichrome. All groups exposed to Ova (OVA, ROO, and OOR) displayed an increase in subepithelial fibrosis that was accompanied by an increase in airway collagen deposition compared to the SAL control group (Figure 5). In fact, a two-fold increase in the amount of basement membrane associated-collagen was observed in the OOR group relative to Ova alone (8.3 ± 0.9 μm vs. 4.2 ± 0.4 μm; p < 0.01). Neither group exposed to RSV alone (RSS and SSR) developed observable airway pathologies.

### Allergen Sensitization of Weanling Mice and Subsequent Infection with RSV Increases the Levels of both Th1 and Th2 Cytokines

**Figure 6:**
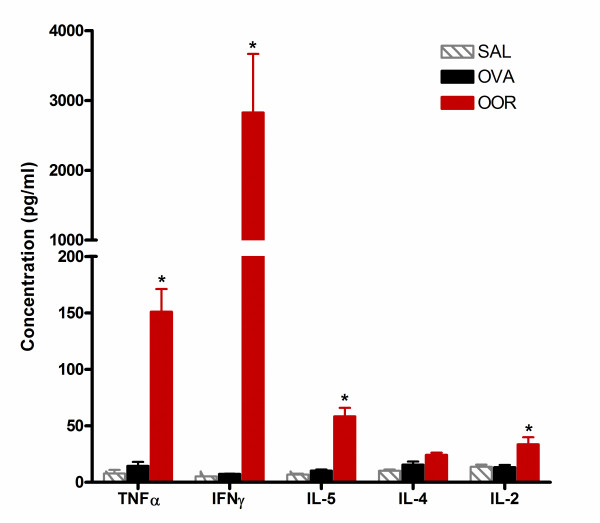
**Th1 and Th2 cytokine levels are elevated in the lungs of OOR mice**. CBA analysis was used to determine the concentration of TNF-α, IFN-γ, IL-5, IL-4, and IL-2 in whole lung homogenates (n = 3 mice per group; *p < 0.01) isolated on protocol day 30. Data are expressed as means ± SEM.

To determine how exposure of weanling mice to Ova and RSV enhanced AHR and led to pulmonary fibrosis, the concentration of various cytokines in whole lung homogenates was measured. When lung homogenates were isolated on the final day of the protocol (i.e., day 34), no significant difference in cytokine levels for TNF-α, IFN-γ, IL-5, IL-4, or IL-2 were observed. In standard Ova models demonstrating allergic inflammation and AHR, expression of Th2 cytokines such as IL-4 and IL-13 typically peak within 48 hours of final allergen challenge and in most cases return to baseline levels within 96 hours[20,22-24]. Therefore, these studies were repeated using whole lung homogenates that were isolated 4 days post infection (i.e., protocol day 4 for the ROO group and day 30 for the OOR group). Cytokine levels from the ROO group were not significantly different from the OVA or SAL groups (data not shown). Interestingly, significantly elevated levels of TNF-α, IFN-γ, IL-5, and IL-2 were observed in the OOR group (151 ± 20 pg/ml, 3663 ± 121 pg/ml, 58 ± 7.5 pg/ml, 34 ± 6.2 pg/ml; respectively) as compared to the OVA group (15 ± 3.4 pg/ml, 7.4 ± 0.12 pg/ml, 10 ± 0.97 pg/ml, 13 ± 1.9 pg/ml; respectively) (Figure 6). Levels of IL-4 in the OOR group were similar to that of the OVA group (24 ± 1.9 pg/ml vs. 16 ± 2.7 pg/ml, respectively).

## Discussion

In the present study, we have shown that exposure of weanling mice to RSV followed by Ova does not lead to  (AHR) or pulmonary pathologies greater than that induced by Ova sensitization and challenge alone. In contrast, exposure of weanling mice to Ova followed by RSV infection leads to long-term pulmonary consequences at the pathophysiological level. RSV infection of weanling mice (unless accompanied by Ova sensitization and challenge) was unable to induce significant histopathologies or AHR. However, early sensitization and challenge with Ova followed by infection with RSV induced a 2.6 fold increase in AHR over SAL controls and a 2 fold increase over Ova alone (Figure 2A). The enhanced AHR coincided with increased: 1) total BAL cellularity (increases were specifically observed in eosinophil and lymphocyte cell numbers); 2) pulmonary inflammation in both the perivascular and peribronchial regions of the lungs; 3) mucus cell hypertrophy and airway mucus production; 4) elevated levels of TNF-α, IFN-γ, IL-5, and IL-2 and airway collagen deposition; and 5) subepithelial fibrosis. These data suggest that pulmonary remodeling events are occurring in weanling mice exposed to allergen followed by RSV infections and, furthermore, that these exposures synergistically enhance pulmonary pathology and physiology. In fact, preliminary data suggest that AHR is prolonged only in the OOR group (> 4 weeks post infection), whereas AHR in the OVA and ROO groups was no longer significantly different from SAL controls forty-eight hours after the last allergen challenge.

Rarely, in humans or mice, is eosinophilia associated with primary RSV infection. However, RSV infection in combination with Ova sensitization and challenge resulted in significant pulmonary eosinophilia in both the pulmonary tissue and BAL fluid (OVA: 1.3 × 10^5 ^± 0.1; ROO: 2.3 × 10^5 ^± 0.3; and OOR: 1.8 × 10^5 ^± 0.2). These results seem inconsistent with those of Peebles et al. [25], who reported decreases in allergen-induced pulmonary eosinophilia when RSV infection preceded allergen challenge. Although the exact cause for this discrepancy is unknown, there are several methodological differences between our studies, which may be pertinent. Peebles and colleagues: 1) used adult mice in their studies, 2) exposed their mice to daily Ova aerosol challenges for eight days, and 3) infected their mice 14 days post Ova challenge. Whereas we: 1) used weanling mice, 2) exposed mice to aerosolized Ova for 20 min for three consecutive days, and 3) infected our mice prior to Ova sensitization (ROO) or immediately following the last Ova challenge (OOR). Although the enhanced recruitment of any one cell did not correlate with pulmonary pathologies, the ratio of total macrophages to eosinophils in the BAL fluid correlated fairly well with pathophysiology (OVA: 0.3 vs. OOR: 0.4).

Culley and colleagues in their neonatal model of RSV infection [13] demonstrated that RSV infection of young mice (0–14 d of age) produced more severe disease initially and upon subsequent rechallenge than did infection of adult mice. In fact, the younger the mouse upon initial RSV infection, the stronger the Th2 polarized immune response to RSV upon secondary infection. Furthermore, human data suggested a link between RSV lower respiratory tract infections in infancy and the later development of asthma [5-10]. Thus, we were expecting to see pulmonary changes in weanling mice (21 d of age) exposed to RSV alone (RSS). However, we failed to see any pathophysiological response to RSV alone (RSS and SSR) or to weanling exposure to RSV followed by allergen (ROO). Although this data was unexpected, we feel that it further supports Culley's original work[13] and the more recent work of Dakhama and colleagues[14] demonstrating the importance of timing of the initial exposure to RSV. Although not presented here, we have preliminary data suggesting that earlier infection with RSV during neonatal development (7 d of age) is sufficient to induce long-term functional consequences in the mouse even in the absence of allergic inflammation. In summary, the age of primary RSV infection has a crucial role in determining disease outcome and suggests that immunity in weanling mice may have matured beyond the polarized Th2 responses of the neonate.

Histological analyses of weanling mice first exposed to Ova and then infected with RSV (OOR group) revealed changes consistent with airway remodeling including subepithelial fibrosis, collagen deposition, smooth muscle hypertrophy, and mucus cell hyperplasia. Histological changes observed in the ROO group were similar to mice receiving Ova alone (OVA group). The amount of airway remodeling observed was positively correlated with increased airway hyperresponsiveness to aerosolized MeCh and with macrophage/eosinophil ratios in the OVA and OOR groups. Our data (presented here and elsewhere[20]) along with the data of others[26] suggest that eosinophils play a prominent role in orchestrating the local pulmonary immune responses and pathologies associated with allergic inflammation (i.e., asthma). It will be interesting to see if the specific loss of eosinophils leads to improvement of pulmonary function parameters and pathologies associated with neonatal RSV infection.

Analysis of cytokine data demonstrated elevations in both Th2 and Th1 cytokines. Previous studies have clearly demonstrated that TNF-α contributes to early clearance of RSV [27]; however, continued production of TNF-α exacerbates RSV-induced illness [28]. Furthermore, neutralization of TNF-α has been shown to reduce clinical illness without an impairment on viral clearance[29]. Interestingly, the OOR group exhibited greater than 10 times the amount of TNF-α observed in any other group. One might also argue that IFN-γ expression in the OOR group, which is upregulated almost 500 fold over the OVA group, is important in the physiologic and histologic injury observed in the OOR group. However, TNF-α levels were still detectable at 17.2 ± 2.4 pg/ml long after (i.e., 8 days) IFN-γ expression was no longer detectable. Though the mechanisms are unclear, the data presented here suggest that the extended upregulation of TNF-α may be important in the lung injury and remodeling events observed in the OOR group.

Interestingly, pulmonary viral titers appeared to be dependent on age at initial RSV infection and allergic phenotype of the mice. Recall that the RSS and OOR groups had significantly lower viral titers than the ROO and SSR groups. We believe that low RSV titers in the RSS group are the result of the Th2-bias that is known to occur in the early neonatal immune system of both mice and humans [30-32], while the low RSV titers in the OOR group are the result of Ova-induced Th2 immune responses. Furthermore, our previous studies demonstrated that eosinophil associated ribonuclease 11 (Ear11) transcripts increase in response to Th2 inflammatory events, such as Ova sensitization and challenge, and that this increase is paralleled by a concomitant increase in RNase activity in the BAL fluid[18]. Ear11 belongs to a family of eosinophil associated RNases, including eosinophil-derived neurotoxin (EDN) and eosinophil cationic protein (ECP). Both EDN and ECP have been shown to possess antiviral activities against a range of single-stranded RNA viruses including RSV [33-35]. Thus, these data cumulatively suggest that the expression of Ear11 in response to a Th2 environment provides the protective (i.e., antiviral) effects observed in the RSS and OOR groups. Ongoing studies in this laboratory with Ear11 depletion strategies are expected to resolve this question.

Severe RSV infections (i.e., requiring hospitalization) during infancy are associated with the development of subsequent wheeze and pulmonary dysfunction including a diagnosis of asthma in later life [6,11,12,36-41]. One hypothesis is that RSV bronchiolitis is simply more severe in atopic individuals. In fact, our studies demonstrated that interactions between RSV infection and allergen-induced immune responses exacerbate pulmonary inflammation and airway physiology. Furthermore, the synergism between RSV and allergen leads to long-term pulmonary consequences such as airway remodeling and may explain progressive lung disease in humans. An alternative hypothesis is that severe RSV infections during infancy are a predisposing factor for the development of airway inflammatory disease states, such as asthma. In this scenario, RSV infection during infancy, when the immune system is still developing and is in a Th2-skewed state, initiates a Th2 polarized primary immune response and subsequently a Th2 polarized memory response to RSV [41]. Such a response may even be heightened in atopic individuals. Although compelling evidence in support of the later hypothesis comes from recently published data [13], we did not find evidence of this in our studies using weanling mice. It is important to point out that this may be due entirely to age at primary infection, since data from other labs[13,14] and unpublished data from our laboratory demonstrate that infections at earlier time points (i.e., 7 days post-partum) leads to long-term immune and pulmonary consequences for the host.

Respiratory tract viral infections account for approximately 85% of asthma exacerbations in children, and 80% of those children have allergic asthma[11,38,39,42,43]; therefore, it is imperative that we understand the mechanisms by which viral infections lead to asthma exacerbations. Although it remains unclear what is responsible for RSV-enhanced allergic inflammation in the lung, what is obvious is that AHR in RSV and Ova exposed mice does not correlate with RSV titer. In contrast, AHR does appear to correlate with increased pulmonary eosinophilia, lymphocyte cell numbers, a decrease in macrophage numbers, and most importantly pulmonary remodeling. Our data suggest that exposure to allergens and RSV leads to increased structural changes in the conducting airways. And it is these structural changes in the developing lung that may be ultimately responsible for the progressive development of the chronic inflammatory disease state known as adult asthma.

## Conclusion

Weanling mice exposed to allergen and RSV exhibit enhanced immune cell responses, which are accompanied by long-term changes in airway structure and function. Although this increased pathologic response was associated with RSV infection, the enhanced pathologies were not dependent upon pulmonary viral titer. Airway remodeling was evident in the lungs of adult mice exposed to RSV and Ova and may provide an explanation for observations suggesting that viral exacerbations of asthma have long-term physiological consequences. Airway remodeling was correlated with elevated levels of TNF-α in the lungs. Extrapolation of these studies to exposures occurring in human neonates highlights the importance of preventative strategies against RSV infection of atopic individuals during neonatal development.

## Abbreviations

RSV, respiratory syncytial virus; AHR, airway hyperreactivity; Ova, ovalbumin; Sal, saline; BAL, bronchoalveolar lavage; BALT, bronchus-associated lymphoid tissue; MeCh, methacholine; Penh, enhanced pause

## Competing interests

The author(s) declare that they have no competing interests.

## Authors' contributions

DB performed mast cell counts in the lung, counted the BAL fluid differentials, performed the invasive pulmonary function studies, and assisted in the preparation of the manuscript. DY determined pulmonary viral titers, performed cytokine assays, and assisted in the preparation of the manuscript. JE collected the airway pathology data, counted the differentials, and assisted in the preparation of the micrographs. DD preformed all of the cell counting and cytospin preparations. SC conceived of the study, performed/assisted in all experiments, and prepared the manuscript. All authors read and approved the final manuscript.
